# Clinical measures of foot posture and ankle joint dorsiflexion do not differ in adults with and without plantar heel pain

**DOI:** 10.1038/s41598-021-85520-y

**Published:** 2021-03-19

**Authors:** Karl B. Landorf, Michelle R. Kaminski, Shannon E. Munteanu, Gerard V. Zammit, Hylton B. Menz

**Affiliations:** 1 Discipline of Podiatry, School of Allied Health, Human Services and Sport, La Trobe University, Victoria, 3086 Australia; 2 La Trobe Sport and Exercise Medicine Research Centre, School of Allied Health, Human Services and Sport, La Trobe University, Victoria, 3086 Australia; 3 Medical Foot Care, 6/230 Blackshaws Road, Altona North, VIC 3025 Australia

**Keywords:** Musculoskeletal abnormalities, Rheumatology, Risk factors

## Abstract

Foot posture and ankle joint dorsiflexion have long been proposed to be risk factors for plantar heel pain, however body mass may be a confounder when investigating these factors. The aim of this study was to determine if clinical measures of foot posture and ankle joint dorsiflexion differ in adults with and without plantar heel pain after accounting for body mass. This was a cross-sectional observational study that compared 50 participants with plantar heel pain to 25 control participants without plantar heel pain who were matched for age, sex and body mass index. Foot posture was assessed using the Foot Posture Index and the Arch Index. Ankle joint dorsiflexion was assessed with a weightbearing lunge test with the knee extended and with the knee flexed. No significant differences (*P* < 0.05) were found between the groups for foot posture, whether measured with the Foot Posture Index or the Arch Index. Similarly, no significant differences were found in the weightbearing lunge test whether measured with the knee extended or with the knee flexed. Clinical measures of foot posture and ankle joint dorsiflexion do not differ in adults with and without plantar heel pain when body mass is accounted for. Therefore, clinicians should not focus exclusively on foot posture and ankle dorsiflexion and ignore the contribution of overweight or obesity.

## Introduction

Plantar heel pain (PHP), also referred to as plantar fasciitis, is one of the most common foot disorders in adults^1^, with population prevalence estimates ranging up to 10%^2,3^. The disorder is also frequently experienced in active groups, including long distance runners^4^. Due to the pain and associated disability related to PHP, people with the condition have been found to have poorer physical function^5^, health-related quality of life^6^ and mental health^7^.

Many risk factors have been proposed for PHP, including intrinsic factors, such as pronated foot posture^8^ and limited ankle joint dorsiflexion^9^. However, a recent systematic review^10^ found that the evidence from studies that have investigated the associations of foot posture and ankle joint dorsiflexion with PHP was inconsistent. Moreover, most studies that have investigated these issues have controlled for age and sex, but they have not controlled for body mass, which has been identified in systematic reviews as being associated with PHP in adults^10,11^. In addition to its association to PHP, body mass has also been associated with foot posture^12,13^ and ankle joint range of motion^14,15^. Therefore, body mass could be a confounding factor. Whether foot posture or ankle joint dorsiflexion are associated with PHP is important because these factors may influence the types of interventions used to treat or even prevent the disorder.

This study aimed to assess whether there are differences in clinical measures of foot posture and ankle joint dorsiflexion between those with and without PHP while controlling for potential confounders. To achieve this aim, the study compared a group of participants with PHP to a group without PHP that were matched for age, sex and body mass index (BMI).

## Results

Seventy-five participants were recruited and completed the study. There were 50 participants with PHP that made up the PHP group and 25 age-, sex- and BMI-matched participants without PHP that made up the control group.

Table 1 presents detailed participant characteristic data, which shows that the two groups were well matched; that is, there were no statistically significant differences in age, sex or BMI. The mean age was 49.1 years in the PHP group and 48.9 years in the control group, with a range for all participants in the study of 23 to 75 years. Women made up 58% of the PHP group and 56% of the control group. The mean BMI was 30.6 kg/m^2^ in the PHP group and 30.2 kg/m^2^ in the control group, with a range for all participants in the study of 20.1 to 47.7 kg/m^2^. In addition, the measure of abdominal obesity (via the waist-hip ratio), education level, number of self-reported medications, and activity levels were all similar between the PHP and the control groups.

**Table 1:** Comparison of participant characteristics between the PHP and control groups—values are means (SDs) unless otherwise stated.

Variable	With PHP group (n = 50)	Without PHP group (n = 25)	Mean difference (95% CI)	*P*-value
Age, years	49.1 (11.6)	48.9 (9.9)	− 0.2 (− 5.6, 5.2)	0.947
Sex-no. of females (%)	29 (58%)	14 (56%)	N/A	0.869^a^
Height, m	1.68 (0.10)	1.73 (0.12)	0.05 (0.00, 0.11)	0.051
Weight, kg	86.1 (17.5)	90.3 (21.4)	4.2 (− 5.0, 13.4)	0.370
BMI, kg/m^2^	30.6 (6.2)	30.2 (7.2)	− 0.4 (− 3.6, 2.8)	0.813
Waist circumference, cm	100.9 (11.4)	101.7 (19.4)	0.8 (− 7.8, 9.3)^b^	0.858^b^
Hip circumference, cm	112.0 (12.6)	111.1 (15.3)	− 0.9 (− 7.5, 5.7)	0.784
Waist-hip ratio	0.90 (0.06)	0.91 (0.09)	0.01 (− 0.03, 0.05)	0.614
Education level-category Median (IQR)	6 (4–7)	6 (5–6.5)	Not applicable	0.785^*c*^
No. of prescribed medications Median (IQR)^d^	0 (0–1)	0 (0–1)	Not applicable	0.430^*c*^
Activity level (kilocalories expended per day)	3745 (1012)	3689 (1034)	56 (− 441, 554)	0.823

^a^*P*-value relates to Chi-squared test.

^b^Mean difference, 95% CIs and *p*-value adjusted as Levene’s test for Equality of Variances was significant (*P* < 0.05).

^*c*^*P*-value relates to Mann–Whitney *U* test.

^d^Variable not normally distributed.

*IQR* Interquartile range.

Regarding duration of symptoms in the PHP group, the median was 6.5 months with a total range of 1.0 to 80.0 months. Their mean first step pain as measured on a 100 mm VAS was 53 mm, pain on the day of their assessment was 39 mm, and pain in the last 7 days was 50 mm.

Table 2 presents the comparisons between the PHP and the control groups for foot posture and ankle joint dorsiflexion. Foot posture and ankle joint dorsiflexion were similar in both groups, and there were no statistically significant differences whether measured with the FPI or the AI. In addition, all differences were either categorised as small or very small when the Cohen’s *d* values were considered.

**Table 2:** Comparison of foot posture and ankle joint dorsiflexion between the PHP and control groups—values are means (SDs) unless otherwise stated.

Variable	With PHP group (n = 50)	Without PHP group (n = 25)	Mean difference (95% CI)	*P*-value	Cohen’s *d*^a^ (category)
**Foot posture**					
Foot Posture Index	4.3 (2.8)	3.4 (3.0)	− 0.9 (− 2.3, 0.5)	0.198	0.32 (small)
Arch Index	0.20 (0.10)	0.21 (0.05)	0.01 (− 0.02, 0.04)	0.554	0.05 (very small)
**Ankle joint dorsiflexion (°)**					
Lunge test-knee extended	32.5 (6.4)	35.5 (6.5)	3.0 (− 0.1, 6.1)	0.054	0.14 (very small)
Lunge test-knee flexed	40.1 (7.8)	41.9 (7.5)	1.8 (−1.8, 5.5)	0.321	0.09 (very small)

^a^Cohen’s *d* values were calculated by dividing the mean between-group difference by the average of the standard deviations for both left and right foot data, and interpretations were taken from Sawilowsky^45^.

## Discussion

This study aimed to assess differences in clinical measures of foot posture and ankle joint dorsiflexion between adults with and without PHP while controlling for important confounders. To achieve this aim, the study compared a group of participants with PHP to a group of participants without PHP that were matched for age, sex and BMI. Participants in the two groups were well matched on these three key matching criteria. Importantly, participants were well matched for BMI, a critical variable in the context of the aim of our study. In addition, the PHP and control groups also had similar abdominal obesity (via the waist-hip ratio), education levels, number of self-reported prescribed medications being taken, and activity levels. Hence, we believe that we minimised potential confounding with the matching criteria that we used.

After determining the participants were well matched, we compared the foot posture of the PHP group with the control group using the clinical measures of the FPI and AI. There was a difference in the mean FPI values between the two groups of less than 1 point (on scale of -12 to + 12), which was not statistically significant and equates to a small difference (i.e. as per the Cohen’s *d* value). The mean FPI values for both groups were 4.3 and 3.4 in the PHP and control groups, respectively, which are indicative of a normal foot posture^16^. In addition, there was a difference in the mean AI values between the two groups of 0.01, which was also not statistically significant and equates to a very small difference. The mean AI values for both groups were 0.20 and 0.21 in the PHP and control groups, respectively, which are indicative of a relatively normal foot posture^17^, although they are on the boundary between high arched and normal arched.

Our finding that foot posture as measured with the FPI does not differ between participants with and without PHP is similar to a cross-sectional study by Sullivan et al.^18^ who compared participants with PHP to control participants without PHP. Like our study, they found no significant difference (*P* = 0.407) in FPI between a PHP group (mean 4.7) and a control group (mean 4.1); the difference being small at 0.6 of a point, which the authors reported as a very small effect size. However, this study did not control for BMI, and as a consequence, the PHP group had a significantly higher BMI (mean 28.8 kg/m^2^ versus 25.6 kg/m^2^, *P* < 0.001).

However, our finding is different to three previous cross-sectional studies that measured foot posture with the FPI. Firstly, Irving and colleagues^19^ compared participants with PHP to age- and sex-matched control participants without PHP. They found the PHP group had a more pronated foot compared with the control group (mean FPI 2.4 versus 1.1, *P* = 0.003, which we calculate to be a medium effect size), and that an FPI ≥ 4 was significantly associated with PHP after adjusting for obesity (BMI ≥ 30 kg/m^2^). However, the FPI cut-off value of 4 was selected based on the distribution of scores in their sample (i.e. the upper quartile) as it preceded the publication of normative values by Redmond et al.^16^.

Secondly, Aranda and Muneura^20^ compared participants with PHP to control participants without PHP. They also found the PHP group had a more pronated foot compared with the control group (mean FPI 6.6 versus 2.8, *P* < 0.001, which we calculate to be a very large effect size). However, the authors did not specifically report participant characteristics for the two groups, so comparisons of important confounding variables were not made. Instead, they simply reported in the methods that, “…inclusion criteria for the control group were patients with characteristics similar to those of the PF group with respect to sex, age, and body mass index (chosen by a pair-matching procedure)…”. Moreover, they did not statistically test whether their matching was successful; they only reported that participants in both groups were in the ‘age range, 19–78 years’. As such, confounding due to these variables cannot be ruled out.

Thirdly, Hogan and co-workers^21^ compared participants with PHP to age-, height-, and weight-matched control participants without PHP. They also found the PHP group had a more pronated foot compared with the control group (mean FPI 6.5 versus 3.5, *P* < 0.020, which we calculate to be a large effect size). However, there are marked differences between the sample recruited by Hogan et al. and that in our study, and the studies mentioned above^18–20^. The mean age in the Hogan et al. study was approximately 26 years, while in our study and the other studies it was substantially older, ranging from 48 to 55 years. The participants in the Hogan et al. study were also relatively active and had a normal BMI (mean BMI approximately 24 kg/m^2^), so they are not representative of a more sedentary middle-aged population with PHP, who make up the majority of cases^22,23^. Accordingly, the findings in the Hogan et al. study can only be generalised to a younger, active population with PHP who are not overweight.

Our finding that foot posture as measured with the AI does not differ between participants with and without PHP is difficult to compare to previous research as only two other cross-sectional studies have used a footprint-based arch index, and they found inconsistent results. The first study by Huang et al.^24^ used an opposing study design to ours. They began by categorising participants according to foot posture (i.e. into a low or a high arched group), and then they assessed for signs or symptoms of PHP. In contrast, all other studies that have evaluated foot posture (including ours) first categorised participants into a PHP or a control group, and then measured foot posture, which is arguably a more valid method. In addition, Huang et al.^24^ used a different arch index to the one we used, which appears to have undergone no validity testing. Nevertheless, they found that participants with low arched feet had a significantly higher rate of PHP, so they concluded that low arched feet were associated with PHP. The two groups were well matched for age, sex and BMI, but they were young adults and had normal BMI (mean age and BMI approximately 30 years and 23 kg/m^2^, respectively). Therefore, the participants in this study were substantially younger and had much lower body mass compared to our study. In contrast to the finding by Huang et al.^24^, the second study by Ribeiro et al.^25^, which used the AI to measure foot posture, found that participants with PHP had a higher arch when compared with a control group. The two groups in this study were reasonably well matched for age, sex and BMI, but again, the sample were runners who were relatively younger with low body mass (mean age and BMI approximately 40 years and 23 kg/m^2^, respectively). This does raise an important issue, however, that PHP may be different (i.e. different aetiology and process) in a younger population compared to an older population—this issue is discussed further in the section below on generalisability.

We also compared ankle joint dorsiflexion of the PHP group with the control group using the weightbearing lunge test with both the knee extended and the knee flexed. There was a difference in the mean ankle joint dorsiflexion with the knee *extended* of approximately 3 degrees, which was not statistically different. Although the difference approached statistical significance (*P* = 0.054), it equates to a very small difference (i.e. as per the Cohen’s *d* value) and is unlikely to be clinically important. In addition, the difference in the mean ankle dorsiflexion with the knee *flexed* was < 2 degrees, which was not statistically significant, nor would it be clinically important as it also equates to a very small difference.

We observed the mean ankle joint dorsiflexion values with the knee flexed to be 40 degrees and 42 degrees for the PHP and control groups, respectively. These values are larger than the mean observed in a population-based study of 1,000 participants that included children and adults—the 1,000 Norms project—which estimated it to be 30 degrees^26^, but slightly smaller than another study in military recruits that recorded a mean of 45 degrees^27^. The mean ankle joint dorsiflexion values we observed with the knee extended were 33 degrees and 36 degrees for the PHP and control groups, respectively, which is slightly smaller than one reliability study of 30 younger adults, which measured a mean of approximately 39 degrees^28^. These differences are likely due to differences in the age of the samples studied and/or measurement techniques used.

Our finding that ankle joint dorsiflexion does not differ between people with and without PHP is difficult to compare to previous cross-sectional studies as findings vary substantially. Four of these studies found that ankle joint dorsiflexion was *less* in participants with PHP compared with those without PHP^18,29–31^, two found *no difference* in ankle joint dorsiflexion^32,33^, and one found *greater* ankle joint dorsiflexion^19^. Moreover, most studies used different methods to ours, making comparison tenuous due to heterogeneity in study methods—most notably, weightbearing versus non-weightbearing assessment of ankle joint dorsiflexion. Indeed, the majority of studies that found *less* ankle joint dorsiflexion in participants with PHP used a passive non-weightbearing assessment^29–31^, where the assessor dorsiflexed the ankle joint, which is arguably a greater source of bias when assessors are not blinded. In contrast, two studies^32,33^ found *no difference* in ankle joint dorsiflexion when comparing participants with PHP to control participants without PHP. Like the studies above, both used a non-weight bearing assessment, although one utilised a passive assessment^32^ and the other an active assessment where the participant dorsiflexed their own ankle^33^. Finally. two studies used a similar assessment technique to our study—a weight bearing ankle lunge test. However, one found *less* ankle joint dorsiflexion^18^ and the other found *greater* ankle joint dorsiflexion in participants with PHP^19^. Most importantly, the majority of studies did not control for BMI. Only one study matched for BMI^31^, while one did not match participants, but instead, adjusted for BMI in the statistical analysis^18^.

There is also the issue of generalisability in three of the previous studies. The control group in the study by Kibler et al.^29^ was not well matched to the PHP group; most of the control group did not engage in long distance running as their source of athletic activity, whereas this was the main activity in the PHP group. In addition, Kibler et al.^29^ and Rome et al.^33^ studied samples who were younger (mean age 31 and 24 years, respectively) and athletic. Likewise, Messier et al.^32^ studied recreational and competitive runners, but did not provide their age. As previously discussed with foot posture, further research investigating whether PHP is different in younger and older populations is warranted—this issue is discussed further below.

Aside from the main findings of our study, it is important to identify if the participants in our study are generalisable to the wider population of adults with PHP. The mean age of participants with PHP in our study was 49 years with a range of 23 to 75 years, 58% were women, and their mean BMI was 30.6 kg/m^2^ with a range of 20.1 to 47.7 kg/m^2^. These values are consistent with other studies from multiple countries that have evaluated adults with PHP in epidemiological investigations^22,23,34^, risk factor studies^10^, and pragmatic randomised trials^35^.

When further assessing BMI, the PHP groups’ values indicate that participants were, on average, obese; although, like other studies, the range of BMIs was large ranging from normal to very severely obese. Regarding symptoms in the PHP group, the median duration of symptoms was 6.5 months in the PHP group with a range of 1.0–80.0 months. Their mean first step pain was 53 mm (measured on a 100 mm VAS), mean pain on the day of their assessment was 39 mm, and their mean pain in the last 7 days was 50 mm, which equates to pain levels that are moderate. Considered together, these findings indicate that the participants in this study are generalisable to the broader population of people with PHP, particularly middle-aged individuals, who make up the majority of cases^22,23^.

As noted previously in the sections above discussing our findings for foot posture and ankle joint dorsiflexion, there is the possibility that there may be two distinct PHP phenotypes in adults; one that is younger and active, and the other that is older, overweight and sedentary. This issue has been raised previously^36^, but evidence for these phenotypes is not currently available, so further research is warranted. In addition, a third phenotype may exist, which is a result of modern western diets and being sedentary, that of younger, obese adults, but again, there is only limited evidence for this from one study^2^.

There are several strengths to our study. Our study matched a general sample of adult participants with and without PHP for BMI. This is important as BMI has consistently been associated with PHP^10,11^, as well as foot posture^12,13^ and ankle joint range of motion^14,15^, so BMI may be a confounding factor in studies like ours. We also utilised valid and reliable assessment methods for foot posture and ankle dorsiflexion. Furthermore, the weightbearing assessment for ankle joint dorsiflexion we used is arguably more clinically relevant compared with non-weightbearing assessments as it more closely reflects load during gait^37^.

Our study also has four limitations that also need to be acknowledged. Firstly, because the sample size for the study was one of convenience (i.e. dictated by limits within the over-arching study), we did not conduct a prospective sample size calculation. Secondly, we chose to recruit participants from the wider adult population with PHP, so our results are generalisable to these people, not to specific sub-populations with PHP. Our findings, therefore, may not apply to younger long-distance runners, for example, who have been found to have a relatively high prevalence of PHP. Thirdly, because cross-sectional studies do not allow the determination of causality, a finding of more pronated or supinated foot posture, or less or more ankle joint dorsiflexion cannot be attributed to causing PHP. Indeed, it could be the other way around, that PHP causes a change in foot posture or ankle joint dorsiflexion, or another confounding variable may lead to change. Only prospective cohort studies can answer this question. Finally, our study, like the other comparable studies, did not utilise blinded assessors, so assessor bias cannot be ruled out. Again, this is a methodological area that can be improved on in future studies, although this will come at the cost of additional research staff and complexity in the study design.

In conclusion, our study found that clinical measures of foot posture and ankle joint dorsiflexion do not differ in adults with and without PHP when body mass is accounted for. Our findings are different to several previous studies, however we controlled for BMI, which many previous studies did not account for. This is important as it appears that BMI most likely influences foot posture and ankle joint dorsiflexion, so previous studies that found an association between these variables and PHP may have been confounded by BMI. Therefore, clinicians should not focus exclusively on foot posture and ankle dorsiflexion and ignore the contribution of overweight or obesity.

## Methods

This study is reported in accordance with the Strengthening the Reporting of Observational Studies in Epidemiology Statement (STROBE)^38^.

### Study design

This was a cross-sectional observational study in people with and without PHP.

### Ethics approval

Ethics approval was obtained from the La Trobe University Human Ethics Committee-Application 14–001. All participants signed written informed consent prior to recruitment into the study.

### Participants

The participants were 75 community-dwelling adults of either sex from the State of Victoria, Australia. There were two groups of participants: (i) a group of 50 participants with PHP, and (ii) a control group of 25 participants without PHP (i.e. in a ratio of 2:1). Participants in the control group were matched to the group with PHP by age (± 5 years), sex, and BMI (± 10%). Participants with PHP needed to have pain underneath the heel (i.e. the inferior or plantar aspect heel), but this was not isolated to a specific point such as the antero-medial aspect of the plantar heel. Participants for this study were also enrolled in an over-arching study that related to medical imaging findings.

### Eligibility criteria

Participants were eligible if they:

i. were aged 18 years or over;
ii. had PHP for at least one month (if recruited to the PHP group);
iii. were able to speak basic English, so they could provide informed consent prior to participation, follow instructions during the project, and to answer questions related to the study accurately.

Participants were excluded from the study if they:

i. had any conditions (e.g. pregnancy, pacemaker, metal fragments, etc.) that would have precluded them from having the medical imaging related to the over-arching study;
ii. had any self-reported inflammatory arthritis (e.g. seronegative arthropathy), endocrine/neurological condition (e.g. diabetic peripheral neuropathy, stroke, etc.), surgery (e.g. amputation, joint fusion, etc.), or trauma (e.g. major fractures) that had affected lower limb sensation or their ability to walk/run.

### Recruitment

Participants were recruited through a variety of means including: advertising posters placed at relevant locations (e.g. La Trobe University, private and public health clinics, sporting and senior citizen clubs), the Health Sciences Clinic at La Trobe University, advertisements on relevant web-sites related to health, direct referral from health care practitioners, and via acquaintances of the investigators involved with the study and snowball sampling. Recruitment began on 12 January 2015 and ended 26 October 2018.

### Sample size

The sample size was one of convenience and was largely dependent on the previously mentioned over-arching study (due to medical imaging costs). The recruitment ratio of 2 participants with PHP to 1 participant without PHP (i.e. controls) was selected to minimise the burden of recruiting age-, sex- and BMI-matched control participants.

### Setting

The study was performed in one of three settings: (i) a research room in the Health Sciences Clinic at La Trobe University in Melbourne, Australia, (ii) a health science clinical tutorial room at La Trobe University, or (iii) in a room at participants’ home with a hard level floor (e.g. linoleum, concrete or wood).

### Protocol

Data were collected by one of three of assessors (authors KBL, MRK, GVZ), all of whom were registered podiatrists with more than 8 years of experience at the time of data collection. Following informed consent, participants were examined in one session that took approximately one to one-and-a-half hours. Data were collected using a standardised assessment form to gather information relating to participant characteristics, medical history including prescribed medications, education, occupation, activities, co-morbidities, foot problems, pain and function, foot posture, footwear, and health status. Using this standardised assessment meant that all participants, whether they had PHP or not, received the same assessment. The assessors were not blinded to whether participants did or did not have PHP, but the standardised assessment was designed to minimise assessment bias by utilising objective measures where possible.

### Data collected

#### General participant characteristics

General participant characteristics were recorded via self-report or assessment. The age of participants at the time of their assessment for the study was recorded in years. In addition, their height in metres (m) and weight in kilograms (kg) was measured; subsequently, their BMI was calculated in kg/m^2^. The normal adult range for BMI as recommended by the World Health Organization (WHO) is 18.5 to 24.9 kg/m^2^, overweight is 25.0 to 29.9 kg/m^2^ and obese is ≥ 30.0 kg/m^2^ ^39^. Further, their waist and hip circumferences were measured (in cm); subsequently, their waist-hip ratio was calculated. The WHO recommend that if the waist-hip ratio is > 0.90 for men and > 0.85 for women, this indicates abdominal obesity^40^.

Participants were also asked to list any prescribed medications they were currently using and report their highest level of education they had completed, which was categorised as: (i) no formal, (ii) less than primary school, (iii) primary school completed, (iv) high school (or equivalent) completed, (v) Technical and Further Education (TAFE) completed, (vi) college/university completed, (vii) post graduate degree completed, (viii) don’t know, (ix) other. In addition, pain levels were measured on a 100 mm Visual Analogue Scale (VAS). Activity level was measured with the Stanford Physical Activity Questionnaire^41^, which calculates the number of kilocalories expended per day.

#### Foot posture

Participants had their foot posture measured using the Foot Posture Index-6 (FPI) and Arch Index (AI). Both measures are used in practice and research environments as valid clinical measures of foot posture.

The FPI-6 uses six criterion-based observations, which are each scored on a 5-point scale (range from − 2 to + 2); these are then summated to produce a final score, which ranges from − 12 (very supinated) to + 12 (very pronated)^42^. The range that represents normal adult foot posture is + 1 to + 7^16^. The FPI-6 is widely used and has been shown to be a valid and reliable measure of foot posture^42,43^.

The AI was calculated using a static footprint obtained using PressureStat carbon paper (FootLogic Inc., South Salem, NY, USA) taken with the participant standing in relaxed bipedal stance. The AI represents the ratio of the area of the middle third of a footprint (i.e. the arch area) to the entire footprint area, excluding the digits^44^. Areas were generated using a Wacom Intuous Graphics Tablet with stylus (Wacom Co. Ltd., Saitama, Japan) and Canvas 11 graphics software (Canvas GFX, Inc, Boston, MA, USA), and the final index calculated in an Excel Spreadsheet (Microsoft 365, Microsoft, Redmond, WA, USA). The AI has been found to be a valid and reliable measure of foot posture^45^. The lower the arch, the higher the AI^44^. The AI can be categorised into three groups: high arch < 0.21, normal arch = 0.21 to 0.28, and low arch > 0.28^17^.

#### Ankle joint dorsiflexion

Ankle joint dorsiflexion was measured while the participants were weight bearing using a lunge test, both with the knee extended, as per Munteanu et al.^28^, and with the knee flexed, as per Bennell et al.^46^. After a brief warm-up period to educate participants on how to perform the lunge test, they were instructed to maximally dorsiflex each ankle one-at-a-time while weight bearing without the heel raising off the ground. While maintaining this position, the investigator recorded the angle of the anterior middle crest of the tibia to the vertical (to the nearest degree) using a non-digital angle finder (i.e. inclinometer). Tests were repeated until a consistent result was obtained; that is, participants were able to carry out the lunge appropriately and were maximally dorsiflexed. These tests have been shown to have high to excellent intratester and intertester reliability when used by experienced testers^28,46^. The mean ankle joint dorsiflexion with the knee flexed has been estimated to be 30 degrees in a large (n = 1,000) population-based study—the 1000 Norms project—that included children and adults^26^. In younger healthy adults, the range for ankle joint dorsiflexion when measured with the weight bearing lunge test is approximately 39 degrees with the knee extended^28^ and 45 degrees with the knee flexed^27^.

### Data analysis

Data were analysed using IBM SPSS Statistics Version 26.0 (IBM Corporation, Armonk, NY). For categorical data, chi-square tests were used to test for differences between groups. For ordinal data (or non-normally distributed continuous data), Mann–Whitney *U* tests were used to test for differences between groups. For continuous data, mean differences with 95% confidence intervals were calculated and unpaired *t*-tests were used to test for differences between groups. All continuous data were first explored for normality prior to inferential analysis. For continuous variables that produced data for the right and left feet (e.g. FPI, AI, ankle lunge), data from both feet were pooled using Linear Mixed Models, which factor in the correlation between the two feet of an individual (i.e. paired data). To provide an estimation of the size of the differences in foot posture and ankle joint dorsiflexion between the groups, Cohen’s *d* effect size values were calculated for foot posture and ankle joint dorsiflexion by dividing the mean difference between the groups by averaged standard deviations for both left and right foot data, and interpretations were taken from Sawilowsky^47^, which are 0.01 = very small, 0.2 = small, 0.5 = medium, 0.8 = large, 1.2 = very large, and 2.0 = huge.

## Data Availability

The datasets generated during and/or analysed during the current study are available from the corresponding author on reasonable request.
