# Author Correction: Clinical measures of foot posture and ankle joint dorsiflexion do not differ in adults with and without plantar heel pain

**DOI:** 10.1038/s41598-021-98192-5

**Published:** 2021-09-10

**Authors:** Karl B. Landorf, Michelle R. Kaminski, Shannon E. Munteanu, Gerard V. Zammit, Hylton B. Menz

**Affiliations:** 1 Discipline of Podiatry, School of Allied Health, Human Services and Sport, La Trobe University, Victoria, 3086 Australia; 2 La Trobe Sport and Exercise Medicine Research Centre, School of Allied Health, Human Services and Sport, La Trobe University, Victoria, 3086 Australia; 3 Medical Foot Care, 6/230 Blackshaws Road, Altona North, VIC 3025 Australia

Correction to: *Scientific Reports* 10.1038/s41598-021-85520-y, published online 19 March 2021

The original version of this Article contained errors in the Discussion section.

 “The mean ankle joint dorsiflexion values we observed with the knee flexed (32 degrees and 35 degrees for the PHP and control groups, respectively) are similar to that observed in a large (n = 1,000) population-based study that included children and adults—the 1000 Norms project—which estimated it to be 30 degrees^26^. Other studies have found the mean ankle joint dorsiflexion with the knee extended to be larger than in our study at approximately 39 degrees with the knee flexed^27^ and approximately 45 degrees knee extended^28^, but the samples in these studies were younger healthy adults.”

now reads:

“We observed the mean ankle joint dorsiflexion values with the knee flexed to be 40 degrees and 42 degrees for the PHP and control groups, respectively. These values are larger than the mean observed in a population-based study of 1,000 participants that included children and adults—the 1,000 Norms project—which estimated it to be 30 degrees^26^, but slightly smaller than another study in military recruits that recorded a mean of 45 degrees^27^. The mean ankle joint dorsiflexion values we observed with the knee extended were 33 degrees and 36 degrees for the PHP and control groups, respectively, which is slightly smaller than one reliability study of 30 younger adults, which measured a mean of approximately 39 degrees^28^. These differences are likely due to differences in the age of the samples studied and/or measurement techniques used.”

The original Article has been corrected.

